# Cerebellar Abnormalities on Proton MR Spectroscopy and Imaging in Patients With Gluten Ataxia: A Pilot Study

**DOI:** 10.3389/fnhum.2022.782579

**Published:** 2022-05-17

**Authors:** Vishwa Rawat, Ritu Tyagi, Inder Singh, Prasenjit Das, Achal Kumar Srivastava, Govind K. Makharia, Uma Sharma

**Affiliations:** ^1^Department of NMR and MRI Facility, All India Institute of Medical Sciences, New Delhi, India; ^2^Department of Neurology, All India Institute of Medical Sciences, New Delhi, India; ^3^Department of Pathology, All India Institute of Medical Sciences, New Delhi, India; ^4^Department of Gastroenterology and Human Nutrition, All India Institute of Medical Sciences, New Delhi, India

**Keywords:** gluten ataxia, cerebellar abnormalities, MR spectroscopy, magnetic resonance imaging, celiac disease, cerebellum, cerebrum

## Abstract

Gluten ataxia is a rare immune-mediated neurological disorder caused by the ingestion of gluten. The diagnosis is not straightforward as antibodies are present in only up to 38% of patients, but often at lower titers. The symptoms of ataxia may be mild at the onset but lead to permanent damage if remain untreated. It is characterized by damage to the cerebellum however, the pathophysiology of the disease is not clearly understood. The present study investigated the neurochemical profile of vermis and right cerebellum and structural changes in various brain regions of patients with gluten ataxia (*n* = 6, age range 40–65 years) and compared it with healthy controls (*n* = 10, 40–55 years). Volumetric 3-D T1 and T1-weighted magnetic resonance imaging (MRI) in the three planes (axial, coronal, and sagittal) of the whole brain and single-voxel ^1^H- magnetic resonance spectroscopy (MRS) of the vermis and right cerebellum were acquired on 3 T human MR scanner. The metabolite concentrations were estimated using LC Model (6.1–4A) while brain volumes were estimated using the online tool volBrain pipeline and CERES and corrected for partial volumes. The levels of neuro-metabolites (N-acetyl aspartate + N-acetyl aspartate glutamate, glycerophosphocholine + phosphocholine, and total creatine) were found to be significantly lower in vermis, while N-acetyl aspartate + N-acetyl aspartate glutamate and glycerophosphocholine + phosphocholine was lower in cerebellum regions in the patients with gluten ataxia compared to healthy controls. A significant reduction in the white matter of (total brain, cerebellum, and cerebrum); reduction in the volumes of cerebellum lobe (X) and thalamus while lateral ventricles were increased in the patients with gluten ataxia compared to healthy controls. The reduced neuronal metabolites along with structural changes in the brain suggested neuronal degeneration in the patients with gluten ataxia. Our preliminary findings may be useful in understanding the gluten-induced cerebral damage and indicated that MRI and MRS may serve as a non-invasive useful tool in the early diagnosis, thereby enabling better management of these patients.

## Introduction

Gluten sensitivity-related disorders have many immune-mediated clinical manifestations such as enteropathy, dermatopathology, and neurological abnormalities (Marsh, 1995). Gluten ataxia (GA) is the most typical neurological disorder with serological evidence of gluten sensitivity, i.e., the presence of anti-gliadin antibodies (AGA IgA and AGA IgG) (Hadjivassiliou et al., 2018). It accounts for up to 40% of the cases of idiopathic sporadic ataxia and 20 % of all ataxias, with the prevalence of <0.001 in the Asia-pacific region (Hadjivassiliou et al., 1998; Ashtari et al., 2019). A strict gluten-free diet provides an improvement in the symptoms of GA (Hadjivassiliou et al., 2017). GA is characterized by damage to the cerebellum due to the widespread deposition of transglutaminase antibodies around the brain vessels. The pathophysiology of neurodegeneration in GA is not clearly understood. However, it has been reported that gluten-mediated neurological pathogenesis may occur due to deposition of immune-complex, cross-reaction of antibodies, and direct neurotoxicity, leading to an inflammatory response in the nervous system (Zelnik et al., 2004; Bushara, 2005; Abenavoli, 2010; Parisi et al., 2015). It has been reported that the antibodies generated against gliadin make the gut leaky and also cross-react with Purkinje cells in the cerebellum causing their irreversible depletion (Hadjivassiliou et al., 2016). Lymphocytic infiltration of the dorsal columns and even peripheral nerves has also been documented (Fitzsimmons et al., 2010).

The diagnosis of GA is not straightforward as antibodies are present in only up to 38% of the GA patients. In the rest of the patients, low titers (undetectable levels) make the diagnosis difficult (Benson et al., 2013). Furthermore, it has also been reported that gluten sensitivity can also be manifested without enteropathy (Aziz et al., 2012), rendering the diagnosis of such GA patients difficult. The symptoms of GA include gait ataxia, dysarthria, upper limb/lower limb ataxia, and ocular signs (Hernandez-Castillo et al., 2016). However, the onset of GA is usually not apparent, but it can rapidly progress, mimicking paraneoplastic cerebellar degeneration (Zis and Hadjivassiliou, 2019). Moreover, the symptoms may be mild at the outset, but they may lead to permanent damage if they remain untreated. Hence, there is a need to understand the pathophysiology of GA and identify non-invasive biomarker/s that may provide an early indication of gluten-induced cerebral damage and treatment management.

Magnetic resonance (MR) based techniques like magnetic resonance imaging (MRI) and MR spectroscopy (MRS) are non-invasive. They play an important role in elucidating the pathophysiology of various brain pathologies (Sharma and Jagannathan, 2004; Symms et al., 2004; Oz et al., 2014; Baldarçara et al., 2015). Of these, MRI provides anatomical images in multiple planes enabling tissue characterization and volumetric measurements. Several structural MRI studies have shown the potential for assessing atrophy patterns in different brain regions in hereditary and spinocerebellar ataxia (Vavla et al., 2018; Deelchand et al., 2019; Hernandez-Castillo et al., 2019; Cocozza et al., 2020). It has been suggested that volumetric measurements can evaluate the disease progression better than the clinical scores (Baldarçara et al., 2015; Vavla et al., 2018; Deelchand et al., 2019; Hernandez-Castillo et al., 2019; Cocozza et al., 2020). Hadjivassiliou et al. (2019) have investigated the correlation between antibodies to Transglutaminase 6 (TG6) and neurological deficits in patients with celiac disease using volumetric MRI. They suggested a link between brain atrophy and autoimmunity to TG6 in these patients (Hadjivassiliou et al., 2019). A review of MRI studies of GA patients recently reported that these patients usually have cerebral atrophy with particular involvement of the cerebellar vermis (Currie et al., 2013a,b). However, in addition to the cerebellum, 40% of the patients with GA have sensory (cerebrum) ataxia rather than cerebellar ataxia and showed no cerebellum atrophy on MRI (Bürk et al., 2001).

*In-vivo* proton magnetic resonance spectroscopy (^1^H MRS) is a valuable technique for assessing the neurochemical abnormalities associated with cerebral damage. The MRS has been widely used to investigate the pathophysiology of various neurological conditions such as Parkinson's disease, dementia, and ataxia (Hadjivassiliou et al., 1998; Firbank et al., 2002; Graff-Radford et al., 2014; Ashizawa et al., 2018). The potential use of MRS in assessing surveillance of patients with paraneoplastic cerebellar degeneration by measuring temporal changes in the level of N-acetyl aspartate (NAA) was also reported (Hadjivassiliou et al., 2013). However, only a few MRS studies in the literature characterize neurochemical abnormalities in patients with GA by a single research group (Wilkinson et al., 2005; Hadjivassiliou et al., 2017). *In-vivo* MRS study by Wilkinson et al. has reported significant differences in the neurochemical profile of NAA and NAA/choline ratios in the cerebellum of patients with GA compared to healthy controls. The authors have concluded that cerebellar neuronal physiology is altered in GA patients even in the absence of cerebellar structural deficit (Wilkinson et al., 2005). Recently, Hadjivassiliou et al. (2017) evaluated the effect of a gluten-free diet on MRS of the cerebellum in patients with GA and found an increase in NAA/creatine ratio after gluten-free diet compared to baseline. These studies have reported relative ratios of neurochemicals. However, there is a lack of data on the absolute measures of neurochemicals in patients with GA. Though a single research group has reported few brain MRI and MRS studies in the Caucasian population in the literature, there is a lack of such a study understanding the neurochemical changes and volumetric brain changes in the patients with GA in the Indian population.

It has been reported that MRS findings can be dependent on population characteristics, ethnicity or race and can change according to the changing environment and racial characteristics (Isamah et al., 2010; Chee et al., 2011). Furthermore, the difference in brain size has been reported between the Indian and Caucasian populations (Sivaswamy et al., 2019). Therefore, the present study investigated the structural changes in the brain using MRI and absolute concentration of neurochemicals (cerebellum and vermis) in GA patients and healthy controls using *in-vivo* MRS to get an insight into the pathophysiology and to identify the putative non-invasive biomarker/s for early indication of gluten-induced cerebral damage in Indian population.

## Patients and Methods

### Patients

The study was conducted in well established Ataxia Clinic, Department of Neurology in collaboration with Celiac Disease (CeD) Clinic, Department of Gastroenterology, All India Institute of Medical Sciences (AIIMS), New Delhi, India. Ataxia patients (both sporadic and familial) positive for serological assays forms part of this study group. We enrolled six right-handed (five males and one female) patients (mean age 50.16 ± 9.28 years) and age range 40–65 years. They were clinically and genetically diagnosed with cerebellar ataxia (CA). As a part of the routine genetic testing, they were tested using standard laboratories protocols for the ataxia-causing known genes in India—SCA1, SCA2, SCA3, SCA7, and SCA12. Two were found to be genetically positive, 2 had a positive family history of ataxias, one had idiopathic sporadic ataxia and one did not undergo genetic testing. Ten healthy, age and ethnically matched controls were also included in the study (mean age 42.7 ± 9.12 years) and age range 30–55 years. At the time of their recruitment, none of the healthy controls had any neurological and genetic illness, nor they were on any treatment with medications active on the central nervous system. The study was approved by Institute Ethics Committee, AIIMS, New Delhi, and written informed consent was obtained from all patients and controls.

### Screening of Patients for Gluten Sensitivity

Four milliliters of blood were drawn from each participant for serological assays. For measuring concentration following commercially available ELISA kits were used: IgA anti tTG ab by QUANTA Lite® h-tTG IgA kits. The AGA IgA and AGA IgG by QUANTA Lite®Gliadin IgA II and QUANTA Lite® Gliadin IgG II kits respectively and TG6 by kits procured from ZEDIRA GmbH, Darmstadt, Germany. The ELISA was done in duplicate in all the sera samples as per the manufacturer's instructions. Positive and negative controls were used. For the study serum titer of >4 U/ml, >20 U/ml, and >41 U/ml were considered positives for IgA anti-TG2 Ab, AGA (IgA and IgG), and anti-TG6 Ab, respectively. Patients were considered gluten sensitive if their serum was found with circulating antibodies.

### Magnetic Resonance Imaging

All sixteen subjects included in the study underwent MR Imaging, on a 3T whole-body MR system (Philips, Ingenia) with a 32-channel head coil. Subjects were positioned supine with their heads supported and immobilized within the head coil using foam pads to minimize head movement and gradient noise. After localization, T1_−_weighted images were obtained in three planes using Magnetization-Prepared Rapid Gradient Echo (MPRAGE) sequence with the following parameters: matrix size = 320 × 318, slice thickness = 0.9 mm, field of view (FOV) = 240 × 240 mm, TR = 10 ms, TE = 4.7 ms, voxel size = 0.75 × 0.75 × 0.75. T2 weighted images were obtained with 30 interleaved 5-mm thick slices without any interslice gap (TE) = 80 ms, TR = 2,676 ms, matrix size = 436 × 357, FOV = 240 × 240 mm, flip angle = 90 degrees, voxel size = 0.55 × 0.65 × 5 mm^3^. The T1 weighted images provide a better contrast-to-noise ratio in white matter regions and are used for the segmentation. The T2-weighted images are good for demonstrating pathology, discriminating structural differences in fluid-filled regions, and important for tracking long-term disease progression (Braga et al., 2012). No contrast agent was administered either for T1 or T2 imaging.

#### Structural MRI Data Processing

3D T1 imaging was done and the digital imaging and communications in medicine (DICOM) images were converted into neuroimaging informatics technology initiative (NIfTI) file format. In all 16 subjects the segmental volumetric calculation of the whole brain and cerebellar structures was performed by uploading the file in NIfTI format to VolBrain and CERES pipeline (Manjón and Coup, 2016). volBrain (http://volbrain.upv.es), and CERES are open-access pipeline, in which pre-processing of the images is based on spatially adaptive non-local means denoising, rough inhomogeneity correction, affine registration to MNI space, fine SPM based inhomogeneity correction and intensity normalization. Here the quality of input images is improved and the images are set into a specific geometry and intensity space. After pre-processing, the images are segmented according to tissues of interest. Segmentation includes non-local intracranial cavity extraction (NICE), tissue classification, non-local hemisphere segmentation (NABS) and non-local subcortical structure segmentation. CERES is mainly used for the segmentation of sub cerebellar structures.

### *In-vivo* MRS

For volume-localized ^1^H-MR spectroscopy first the scout images, T1 weighted images, and multislice T2-weighted images in the axial, coronal and sagittal planes of the whole brain were acquired using a standard spin-echo pulse sequence. Using the reference MR images, single-voxel proton MR spectra were acquired using the point-resolved spin-echo pulse sequence (PRESS) with the following parameters: TR = 2000 ms; TE = 30 ms; TA = 9 minutes 8 s; the number of averages (NA) = 256. We have used short TE for MRS as it offers a higher signal-to-noise ratio (SNR), improved detection and quantification of molecules with short T2 such as myoinositol (mI), glutamine/glutamate (Glx), due to less relaxation-induced signal loss (Cianfoni et al., 2011). The disadvantage associated with short TE is incorrect baseline determination which may lead to an artifactual elevation in the determination of concentration (Brief et al., 2005). On the contrary, it is easier to correct the baseline in the spectrum acquired with long TE, however, it provides information on a lesser number of metabolites (Brief et al., 2005). Further, Cr has a significantly shorter T2 relaxation time compared to metabolites like NAA and Cho, which leads to incorrect estimation of relative (metabolite/Cr) and absolute metabolite concentrations in a long TE MRS without applying T2 correction (Brief et al., 2005).

In order to optimize the magnetic field both global and voxel shims were carried out. Automated global shimming minimizes the B_0_ inhomogeneities while localized voxel shimming further minimizes the B_0_ field variations over the voxel of interest. The spectra were acquired from right cerebellum and vermis with voxel size of 15 mm × 15 mm × 15 mm and care was taken to minimize the inclusion of cerebrospinal fluid within the prescribed spectroscopic volume for methodological consistency. The cerebellar vermis has a serpentine or wormlike shape and it varies slightly according to the individual. The MRS studies reported in the literature on patients with ataxia and GA have used the varied voxel size of vermis in the range of 20 mm × 20 mm × 20 mm (Lirng et al., 2012), 25 mm × 10 mm × 25 mm (Adanyeguh et al., 2015), 20 mm × 10 mm × 20 mm (Currie et al., 2013b), 15 mm × 15 mm × 15 mm (Guerrini et al., 2009). A width of 15 mm of the voxel in vermis may have minor contributions from the surrounding areas of the vermis in our study.

The line-width after voxel shim ranged from 5 to 14 Hz depending on the region studied. Additionally, a spectrum of the same voxel without water suppression was also acquired and the unsuppressed water signal was used as an internal reference to estimate the metabolite concentrations. The absolute concentration of metabolites was determined using an (LC Model) user-independent, frequency domain fitting program, version 6.1-4A, with a basis set of model metabolites. An eddy-current correction was also carried out. The errors in the determined metabolite concentration are expressed in the percent standard deviation (%SD) of the concentration and represent the 95% confidence interval of the estimated concentration. In the present study, we have considered the absolute metabolites values when the Cramer-Rao lower bound or the LC model concentrations were below 20%, indicating that these metabolites could be reliably estimated. The concentration of metabolites was expressed as institutional units related to millimoles per kg (mM/kg).

### Segmentation

3D T1-weighted images acquired with magnetization-prepared rapid gradient echo (MPRAGE) sequence were used to carry out segmentation of tissue subtypes in the SVS voxel using the segment-editor module of an open-source neuroimaging package 3D-Slicer (Fedorov et al., 2012). Screenshots of the exact position of the SVS voxel on MR images in all the axial, sagittal, and coronal slices were captured on the MR scanner and were used as voxel prescription images. A mask representing the 3D SVS voxel was then created manually using these screenshots as a reference. The mask was then co-registered to the DICOM image at the exact position as the actual 3D SVS voxel in the voxel prescription images and fractions of white matter (WM), gray matter (GM), and cerebrospinal fluid (CSF) were determined using the segmentation module.

### Partial Volume Correction for Metabolite Concentration

The concentration of metabolites was then corrected for the amount of CSF using the following equation given by (Quadrelli et al., 2016).


$$\begin{array}{l} C=C_{0}\;\left[1/\left(1-V_{\text{CSF}}\right)\right] \end{array}$$

where,

C = corrected metabolite concentration;

C_0_ = metabolite concentration obtained through LC Model;

V_CSF_ = volume fraction of CSF contained within the SVS voxel;

We have also calculated the weighted measures (Wc) of the concentration of metabolites according to the relative proportion of gray matter (GM) and white matter (WM) following the procedure given in the literature (Mato Abad et al., 2014) for white matter changes in patients with GA and for their better discrimination. The equation used for the determination of Wc is given below (Mato Abad et al., 2014):


$$\begin{array}{l} Wc=LC_{\text{c}}\cdot P\left(M_{\text{c}}\right) \end{array}$$

Where, Wc = Weighted measure; LC_c_ = Concentration of metabolite from LC Model; P(Mc) = Theoretical distribution of metabolites in a particular voxel according to the relative proportions of GM, WM, and CSF.

P(Mc) was determined as follows:


$$\begin{array}{l} P\left(Mc\right)=P\left(GM\right)\cdot P\left(Mc|GM\right)+P\left(WM\right)\cdot P\left(Mc|WM\right)\\ +P\left(CSF\right)\cdot P\left(Mc|CSF\right) \end{array}$$

Where,

P(GM), P(WM), and P(CSF) are, respectively, the proportion of GM, WM, and CSF inside the voxel.

P(Mc|GM), P(Mc|WM) and P(Mc|CSF) are the probabilities of the metabolite concentration given this volume of GM, WM, and CSF (Mato Abad et al., 2014).

Thus, Wc is a measure of the concentration of metabolite which was corrected for the theoretically expected ratio of metabolite and the atrophy of volume within a voxel (Mato Abad et al., 2014). The concentrations of metabolites published in the literature (Tal et al., 2012; Bustillo et al., 2017) were used to obtain the contribution of GM and WM to the quantity of metabolites. The contribution of CSF to metabolite concentration was considered negligible (Hetherington et al., 2001).

### Statistical Analysis

The percentage of different brain volumes and the concentration of different neurochemicals were calculated for GA patient and HC group and presented as mean ± standard deviation. A student t-test was used to evaluate the difference between patients and controls for ^1^H MRS and volumetric data using the SPSS 20.0 software (SPSS Inc. Chicago, IL, USA). The level of significance was set at *p* < 0.05. The Pearson correlation was used to calculate the correlation of the concentration neurochemicals and the volumetric changes in the brain of GA patients.

## Results

The demographic characteristics of GA patient group are presented in Table 1. GA patient group consisted of 6 patients (five men, one woman; aged between 40 and 65 years). Of these, four patients had mild (mobilizing independently or with one walking aid), one patient had moderate (mobilizing with two walking aids) while one had severe (wheelchair dependant) ataxia. Control group consisted of 10 healthy volunteers (5 men, 5 women; age range (30–55 years) with no neurological/psychiatric disease/no medical or family history of ataxia.

**Table 1:** Demographic characteristics, clinical manifestations, and serology of patients with Gluten Ataxia.

**Demographic characteristics**	
Number of patients	*n* = 6
Mean age ± SD; (range)	50.2 ± 9.3; (40–65) years
Age of onset (mean ± SD)	46.2 ± 7.1 years
Gender	Male (*n* = 5), female (*n* = 1)
**Clinical manifestations**	
Symptoms	Loss of balance, diarrhea, constipation, weakness
Severity of ataxia	Mild (*n* = 4), moderate (*n* = 1), severe (*n* = 1)
**Serology**	
IgA anti-AGA	Positive (*n* = 4); Negative (*n* = 2)
Mean ± SD	31.76 ± 20.56 (*n* = 4)
Cut off value	>20
Anti-TG 6	Positive (*n* = 1); Negative (*n* = 5)
Concentration	62.1 (*n* = 1)
Cut off value	>41
Anti-tTG	Positive (*n* = 1); Negative (*n* = 5)
Concentration	94.6 (*n* = 1)
Cut off value	>4

*IgA, immunoglobulin A; anti-AGA, antibodies to gliadin; anti-tTG, antibodies to tissue transglutaminase 2; anti-TG 6, antibodies to tissue transglutaminase 6*.

### Comparison of Whole Brain Volume and Brain Parts Between GA Patients and HCs

**Figure 1:**
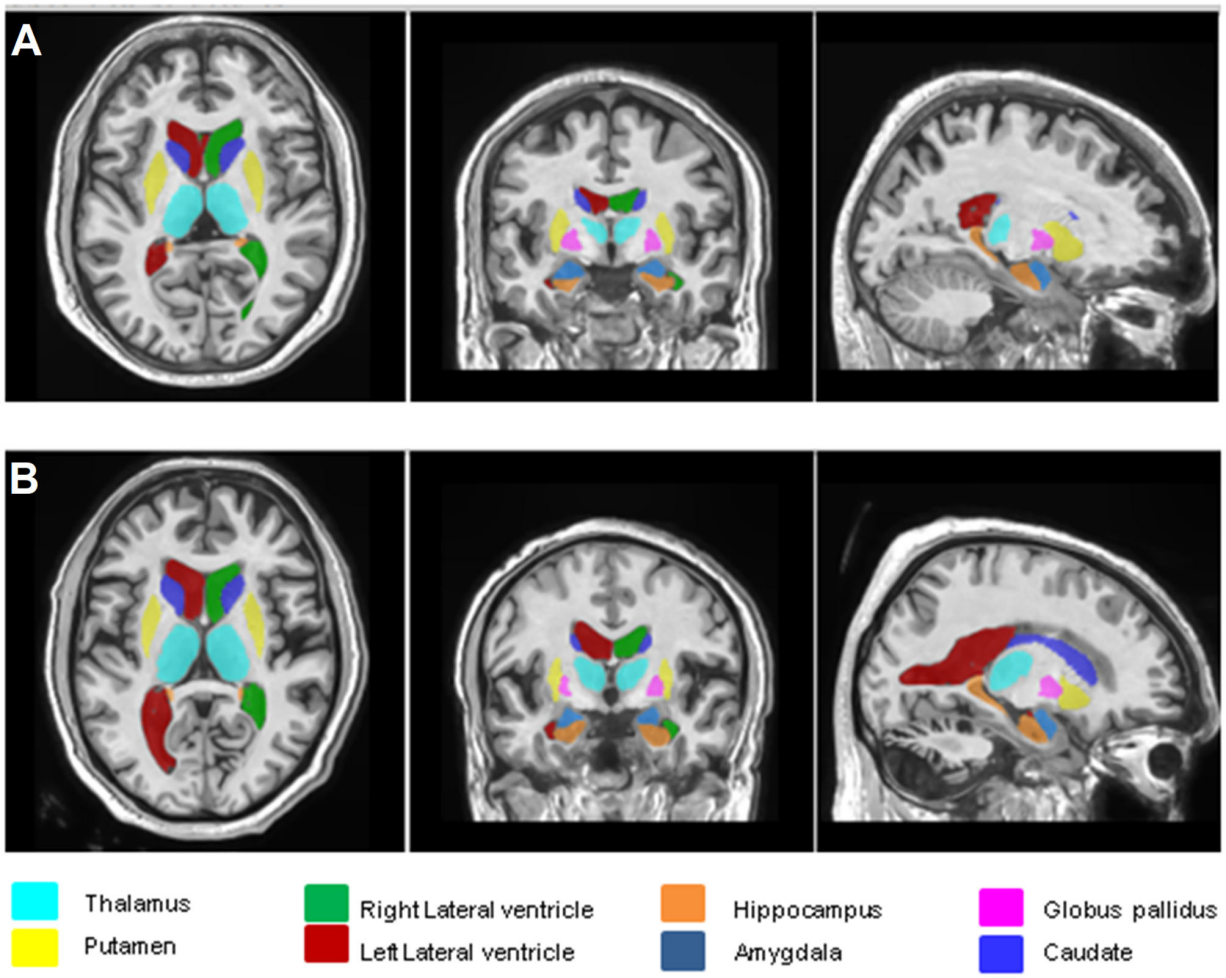
Representative volBrain images of brain and sub-parts obtained from a healthy control (male, 44 years old) **(A)** and a patient with gluten ataxia (male, 55 years old) **(B)**.

**Figure 2:**
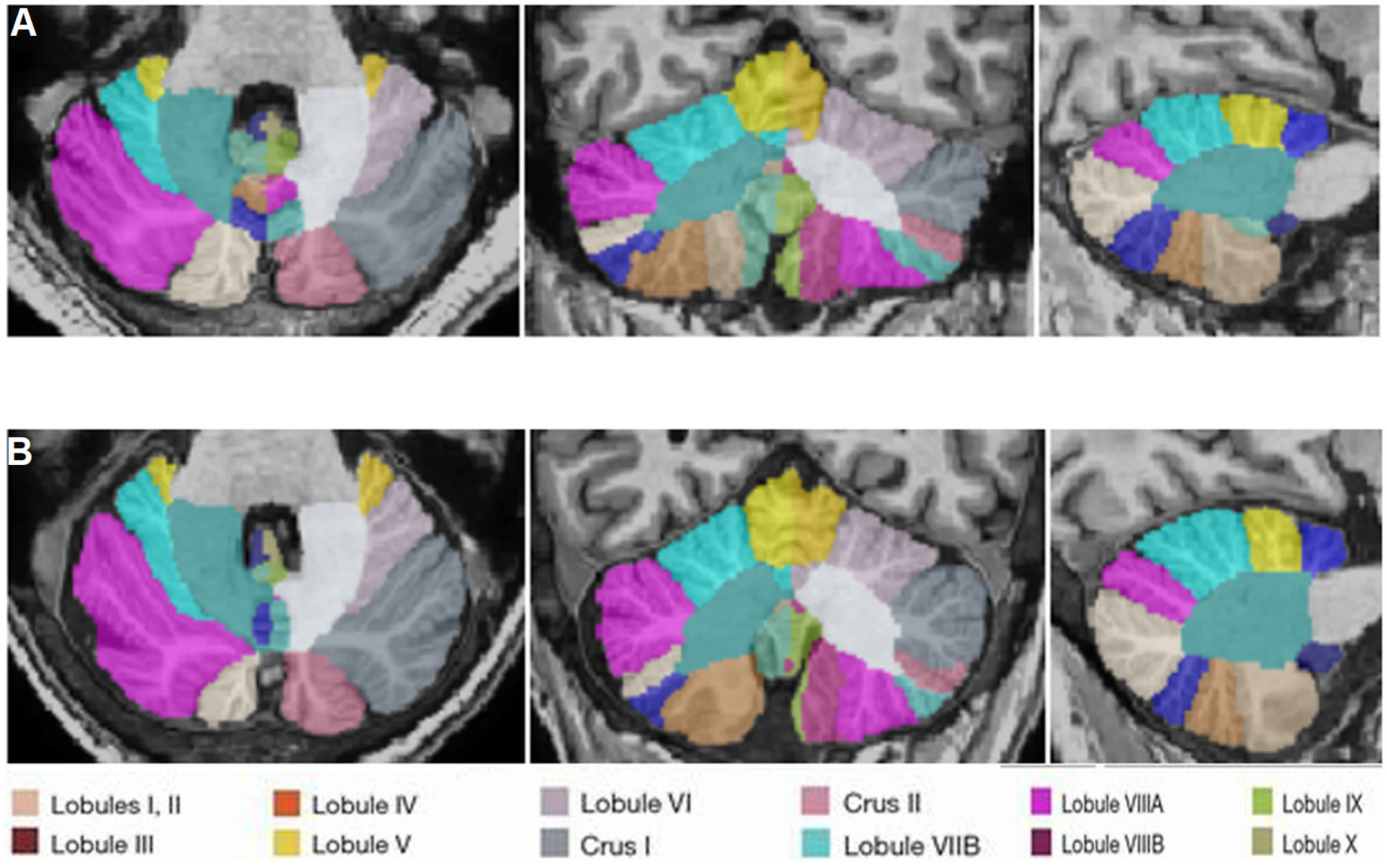
Representative CERES images of cerebellum and lobules taken from a healthy control (male, 44 years old) **(A)** and a patient with gluten ataxia (male, 55 years old) **(B)**.

Figures 1A,B show the representative volBrain images of brain and sub-parts obtained from a 44 years old male healthy control and a patient with gluten ataxia (male, 55 years old), respectively. Figures 2A,B show the representative CERES images showing segmentation of lobules of the cerebellum from a healthy control (male, 44 years old) and a patient with gluten ataxia (male, 55 years old), respectively. The result of the volumetric analysis is reported as % of total intracranial volume (Tables 2, 3). GA patients showed a significant reduction in total brain white matter (WM) (*p* < 0.03), the white matter of the cerebrum (*p* < 0.04), the volume of lateral ventricles (*p* < 0.04) and thalamus (*p* < 0.01) compared to healthy controls (Table 2). GA patients also showed significantly reduced total cerebellar WM (*p* < 0.02) significantly affecting the lobule X (*p* < 0.03) (Table 3). The volume of caudate, hippocampus, putamen, accumbens, globus pallidus, and amygdala were not significantly different between the two groups (Table 2).

**Table 2:** Volumetric analysis of individual brain regions of patients with Gluten Ataxia (GA) and Healthy Controls (HC).

**Regions of interest**	**Volume percentage (mean** **±SD)**		***p* value**
	**GA (*n* = 6)**	**HC (*n* = 10)**	
**Total brain**			
WM + GM	78.64 ± 7.68	84.38 ± 5.53	0.12
WM	31.89 ± 6.65	38.97 ± 4.26	**0.03**
GM	46.75 ± 3.73	45.41 ± 8.86	0.74
CSF	21.35 ± 7.68	15.62 ± 5.53	0.12
**Cerebrum**			
WM + GM	69.57 ± 6.43	73.3 ± 5.21	0.24
GM	40.29 ± 2.45	38.38 ± 8.52	0.60
WM	29.27 ± 5.71	34.92 ± 4.03	**0.04**
**Cerebellum**			
WM + GM	7.65 ± 2.39	9.33 ± 0.65	0.07
GM	6.04 ± 1.93	6.69 ± 0.70	0.38
WM	1.61 ± 0.89	2.91 ± 0.96	**0.02**
**Other regions**			
Caudate	0.51 ± 0.07	0.55 ± 0.12	0.58
Hippocampus	0.56 ± 0.04	0.51 ± 0.12	0.40
Amygdala	0.12 ± 0.02	0.10 ± 0.05	0.34
Lateral ventricles	2.01 ± 1.32	0.88 ± 0.52	**0.04**
Putamen	0.55 ± 0.07	0.62 ± 0.06	0.06
Thalamus	0.74 ± 0.08	0.85 ± 0.06	**0.01**
Accumbens	0.04 ± 0.01	0.04 ± 0.01	0.95
Globus pallidus	0.14 ± 0.02	0.15 ± 0.03	0.42

*WM, white matter; GM, gray matter; CSF, cerebrospinal fluid. Bold values indicate significant p value*.

**Table 3:** Volumetric analysis of individual cerebellar regions of patients with gluten ataxia (GA, *n* = 6) and healthy controls (HC, *n* = 10).

**Region of interest**	**Volume percentage (mean** **±SD)**		***p*-value**
	**GA**	**HC**	
**Cerebellum and lobules**			
Cerebellum	7.82 ± 1.80	8.66 ± 0.31	0.22
Lobule i-ii	0.004 ± 0.002	0.004 ± 0.002	0.55
Lobule iii	0.07 ± 0.019	0.08 ± 0.02	0.42
Lobule iv	0.26 ± 0.05	0.29 ± 0.03	0.13
Lobule v	0.48 ± 0.11	0.49 ± 0.05	0.96
Lobule vi	1.09 ± 0.29	1.09 ± 0.14	0.99
Lobule crus i	1.69 ± 0.43	1.75 ± 0.22	0.72
Lobule crus ii	1.06 ± 0.23	1.11 ± 0.16	0.59
Lobule vii-b	0.54 ± 0.15	0.62 ± 0.07	0.20
Lobule viii-a	0.69 ± 0.189	0.77 ± 0.09	0.33
Lobule viii-b	0.48 ± 0.15	0.54 ± 0.06	0.36
Lobule ix	0.49 ± 0.13	0.50 ± 0.06	0.88
Lobule x	0.06 ± 0.02	0.09 ± 0.02	**0.03**

*Bold values indicate significant p value*.

### Comparison of Neurochemical Profile Between Patients With GA and HCs

**Figure 3:**
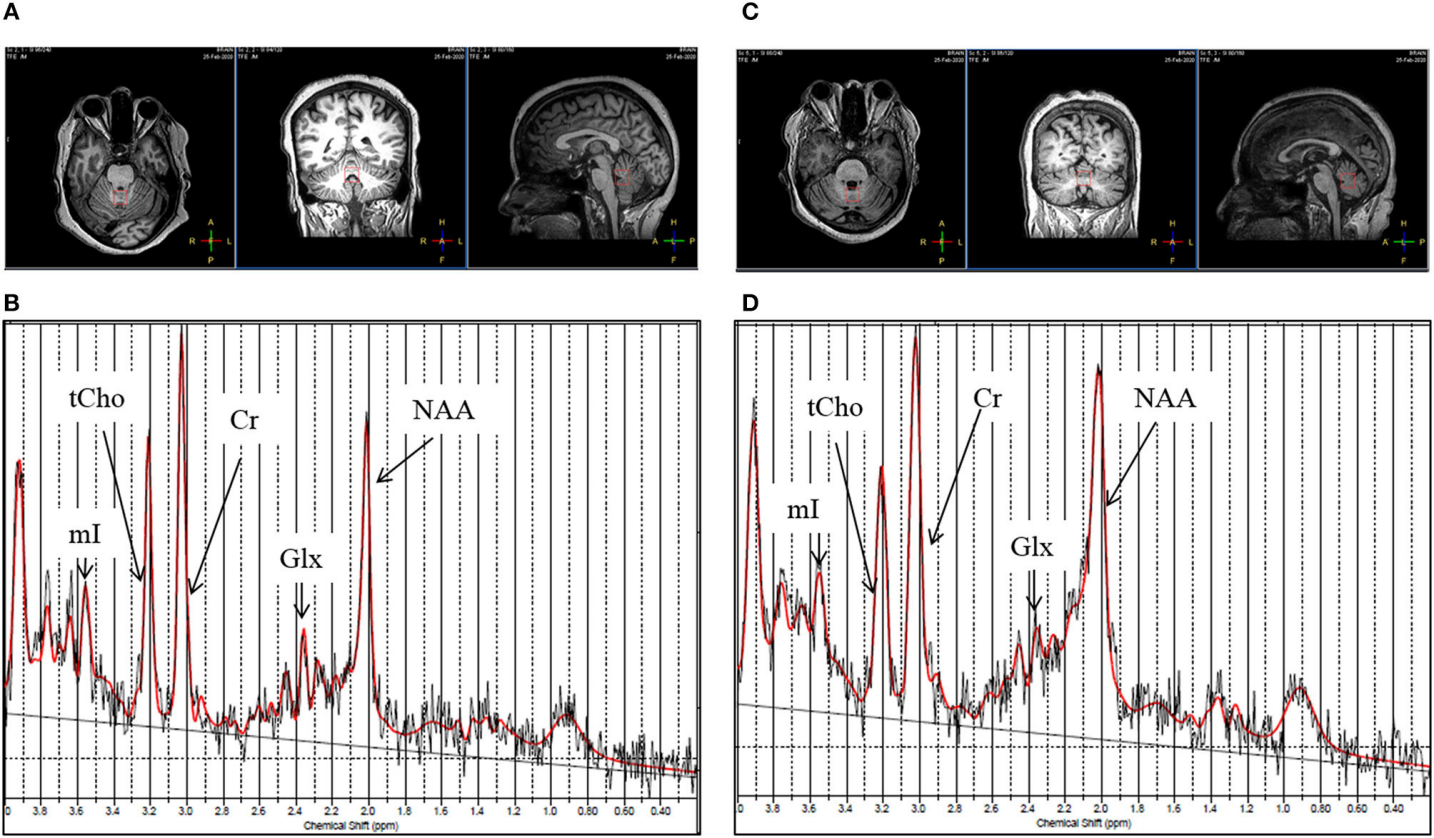
**(A)** T1-weighted images of a healthy control (female, 44 years old) in axial, coronal, and sagittal plane. **(B)** Representative LC Model fitted ^1^H-magnetic resonance spectroscopy spectrum obtained from the vermis of the same healthy control from the voxel highlighted in **(A)**. **(C)** T1-weighted image of a patient with gluten ataxia (female, 50 years old) in axial, coronal and sagittal plane. **(D)** Representative LC Model fitted ^1^H-magnetic resonance spectroscopy spectrum acquired from the vermis of the same patient with gluten ataxia from the voxel highlighted in **(C)**.

**Figure 4:**
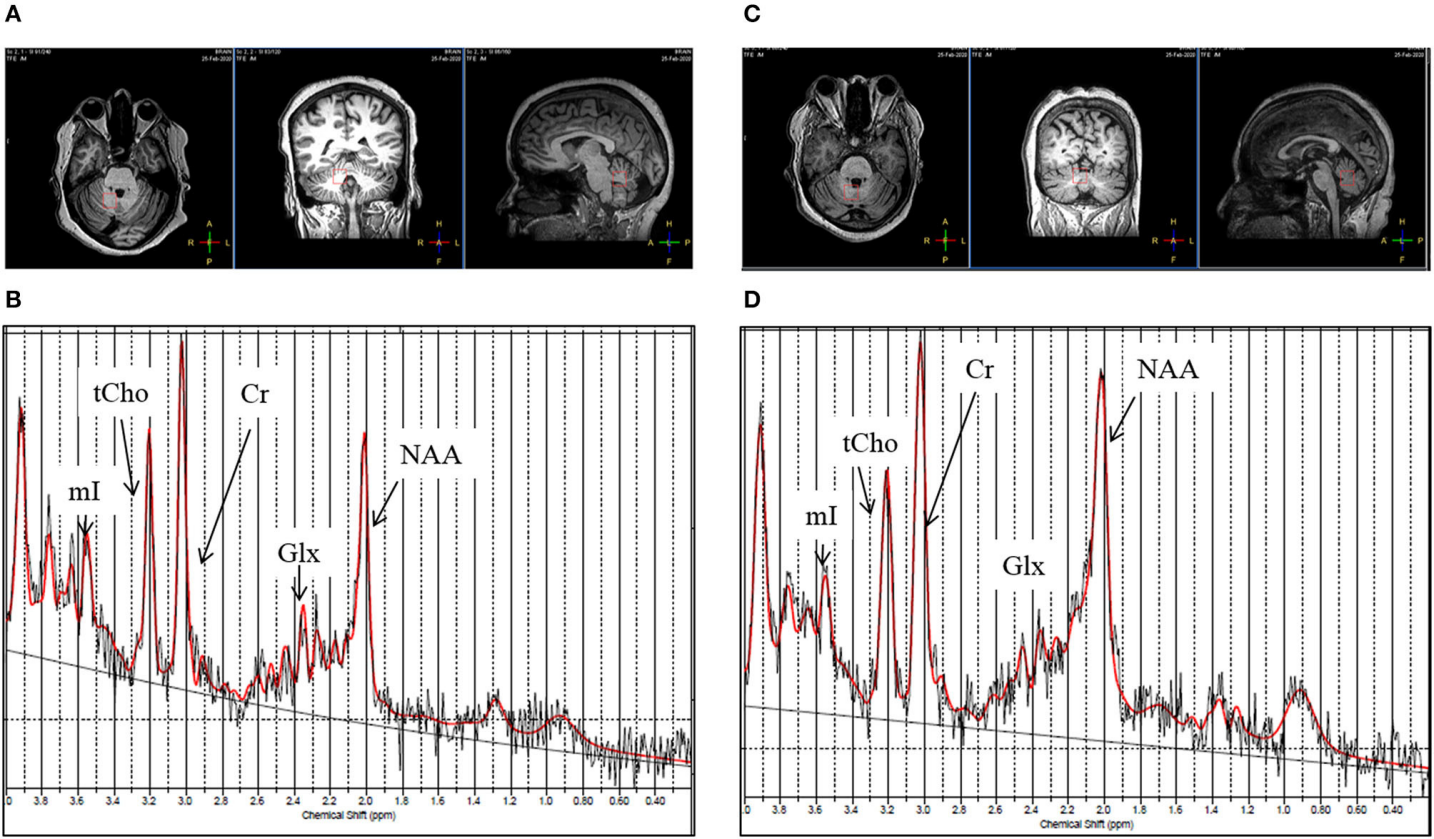
**(A)** T1-weighted images of a healthy control (female, 44 years old) in axial, coronal and sagittal plane. **(B)** Representative LC Model fitted ^1^H-magnetic resonance spectroscopy spectrum obtained from the right cerebellum region from the same healthy control from the voxel highlighted in **(A)**. **(C)** T1-weighted image of patient with gluten ataxia (female, 50 years old) in axial, coronal and sagittal plane. **(D)** Representative LC Model fitted ^1^H-magnetic resonance spectroscopy spectrum acquired from the right cerebellum region of the same patient with gluten ataxia from the voxel highlighted in **(C)**.

Figures 3A–D show the T1-weighted MR images of healthy control (female, 44 years old) and a patient with gluten ataxia (female, 50 years old) in axial, coronal, and sagittal planes and representative LCModel fitted ^1^H-magnetic resonance spectroscopy spectra for vermis acquired from these subjects. The T1-weighted images of the brain in all the three planes and the representative LCModel fitted ^1^H-magnetic resonance spectroscopy spectra for the right cerebellum acquired from the same healthy control and the patient with gluten ataxia are shown in Figures 4A–D. The concentrations (corrected for CSF) of neurochemicals determined using ^1^H-MRS are summarized in Table 4. The patients with GA had statistically significantly reduced concentration of total N-acetyl aspartate (tNAA: N-acetyl aspartate+ N-acetyl aspartate glutamate) (*p* < 0.008) and total choline (tCho: glycerophosphocholine+phosphocholine) (*p* < 0.05) in the vermis. In the right cerebellum, GA patient group showed a statistically significant reduction in the concentration of tNAA (*p* < 0.02). The proportions of gray matter P(GM), white matter P(WM), and cerebrospinal fluid P(CSF) in the MRS voxel for vermis and right cerebellum of GA patients and HCs were not significantly different (Table 5).

**Table 4:** Comparison of the concentrations of neurochemicals (corrected for CSF) and their weighted measures in the vermis and right cerebellum of patients with Gluten Ataxia (GA) (*n* = 6) and Healthy Controls (HC) (*n* = 10).

**Neurochemicals**	**Vermis**		***p*-value**	**Right Cerebellum**		***p*-value**
	**GA**	**HC**		**GA**	**HC**	
**Concentrations [mM/Kg, (Mean** **±SD)]**						
tNAA	5.15 ± 1.25	7.11 ± 0.81	**0.008**	5.75 ± 0.95	7.04 ± 0.67	**0.02**
tCho	1.88 ± 0.46	2.29 ± 0.19	**0.05**	1.97 ± 0.41	1.91 ± 0.13	0.73
tCr	7.53 ± 2.58	8.91 ± 0.95	0.22	7.12 ± 1.86	7.22 ± 1.62	0.91
Glx	11.73 ± 1.37	11.61 ± 2.08	0.91	10.56 ± 2.04	10.58 ± 1.77	0.98
mI	6.97 ± 2.19	6.74 ± 0.91	0.80	6.79 ± 2.59	5.87 ± 0.69	0.38
**Weighted measures (Mean** **±SD)**						
tNAA	2.14 ± 0.91	3.17 ± 0.37	**0.02**	2.50 ± 0.89	3.30 ± 0.33	**0.05**
tCho	0.75 ± 0.24	0.96 ± 0.05	**0.03**	0.83 ± 0.07	0.91 ± 0.05	**0.05**
tCr	2.99 ± 1.18	4.15 ± 0.42	**0.03**	3.02 ± 1.09	3.34 ± 0.53	0.52
Glx	4.72 ± 1.51	5.62 ± 1.04	0.25	3.60 ± 2.49	4.85 ± 0.78	0.23
mI	2.65 ± 0.52	3.03 ± 0.26	0.12	2.74 ± 0.63	2.74 ± 0.27	0.98

*tNAA, total NAA (N-acetyl aspartate+ N-acetyl aspartate glutamate); mI, myoinositol; tCho, total choline (glycerophosphocholine+phosphocholine); tCr, total creatine (creatine+phosphocreatine); Glx, glutamate+glutamine. Bold values indicate significant p value*.

**Table 5:** The proportions (mean ± SD) of gray matter P(GM), white matter P(WM), and cerebrospinal fluid in P(CSF) in the voxel placed during MRS in the vermis and right cerebellum of patients with gluten ataxia (GA) and healthy controls (HC).

**The proportions of tissue sub-type**	**Vermis**			**Right cerebellum**		
	**GA**	**HC**	***p* value**	**GA**	**HC**	***p* value**
P(GM)	0.42 ± 0.29	0.62 ± 0.09	0.10	0.39 ± 0.27	0.42 ± 0.04	0.76
P(WM)	0.38 ± 0.24	0.26 ± 0.06	0.20	0.46 ± 0.18	0.53 ± 0.06	0.41
P(CSF)	0.20 ± 0.18	0.13 ± 0.06	0.30	0.15 ± 0.13	0.05 ± 0.03	0.10

We have also calculated the Wc of the concentration of metabolites according to the relative proportion of gray matter (GM) and white matter (WM) following the procedure given in the literature (Mato Abad et al., 2014) and presented in Table 4. The Wc of the tNAA, tCho and tCr in vermis, while tNAA and tCho were found to be significantly lower in the patients with GA compared to HC (Table 4).

### Correlation of the Concentration of the Neurochemicals and Brain Volumetric Changes

A significant correlation (r = 0.9, *p* = <0.05) was observed between the concentration of tNAA (both cerebellum and vermis) and tCho (vermis) with lobule X of the cerebellum.

## Discussion

In recent years, the developments in neuroimaging techniques such as MRI and MRS have enabled the characterization of the structural and neurochemical changes, elucidating the pathophysiology of various brain diseases (Symms et al., 2004; Oz et al., 2014). Among many types of ataxias, GA is an auto-immune disease caused by the ingestion of gluten in the genetically susceptible individuals (Hadjivassiliou et al., 2014). The non-invasive nature, versatility and possibility of evaluation of multiple parameters in a short time makes the MRI and MRS as an important tool for understanding the disease pathophysiology and identifying disease biomarkers. Additionally, the possibility of repetitive measurements using these techniques can play an important role in the management of diseases like GA that require long term follow-up for assessment of neurological abnormalities. It is critical to understand the pathophysiology of the GA for the early diagnosis and treatment management strategies, however, till date, it was not investigated at many sites using MRI and MRS. To the best of our knowledge there are only two *in-vivo* MRS study and one study using combined MRI and MRS approach in GA patient in Caucasian population by a single research group (Wilkinson et al., 2005; Hadjivassiliou et al., 2019; Zis and Hadjivassiliou, 2019). The present study has evaluated the volumetric and neurochemical changes in the brain in GA patients compared to HCs using MRI and MRS in Indian population. Several interesting observations emerged from our study.

Our results revealed significantly reduced volume of white matter in the total brain, cerebellum and cerebrum of the patients with GA compared to HC. It is known that white matter is an essential component of neural networks and acts as a connecting link between various gray areas in the brain. White matter contains myelin sheathed axons that relay the information between different brain areas. The reduction in the myelin sheathed cells can cause delay both in passing and processing of the information. Delayed or staggered processing of information may result in clumsy and uncontrolled movements which are generally seen in the patients with GA (Vinagre-Aragón et al., 2018). The loss of white matter has been reported in several neurodegenerative diseases. The alterations in the volume of white matter have been found to affect the cognition (Filley and Fields, 2016). Kang et al. (2014) have reported that disruption of white matter integrity was related to the clinical severity of the disease in the patients suffering with SCA3. Correlation of white matter degeneration and severity of ataxia was also found in the patients with SCA type 7 (Kang et al., 2014). In a volumetric MRI study of patients with celiac disease having TG6 autoantibodies, significant atrophy of subcortical brain regions was reported in 40% of the patients. The white matter abnormalities were also reported in these celiac disease patients, which is in agreement with our findings (Hadjivassiliou et al., 2019). Croall et al. (2020) have recently shown white matter damage in patients with celiac disease having indications of worsened mental health and cognitive deficit, compared to the healthy controls.

The significantly reduced volume of white matter in our study might be due to the damage caused by inflammatory response by gluten-sensitive antibodies. It has been reported that the etiology of neurological manifestation of gluten sensitivity has an immunological manifestation (Hernandez-Lahoz et al., 2011). The patients with gluten ataxia have been shown to have positive oligoclonal bands on examination of CSF. Furthermore, the pathological data suggested an inflammatory pathogenesis with evidence of perivascular inflammation with predilection of brainstem, cerebellum and peripheral nerves (Hadjivassiliou and Croall, 2021). Patients with GA have evidence of IgA deposits against tissue transglutaminase on vessels within cerebellum and brainstem. Transglutaminase leads to vascular based inflammation that may results in breakdown of blood brain barrier thus allowing the entry of gluten-sensitive antibodies in brain (Hadjivassiliou et al., 2006), the same antibodies can cross-react with Purkinje cells in the cerebellum causing their irreversible depletion (Hadjivassiliou et al., 2016).

Another interesting observation of our study was significantly reduced volume of lobule X of the cerebellum in the patients with GA compared to HC. The lobule X is also known as flocculonodular lobe and it plays an important role in regulating and orienting the eye movement through vestibulo-ocular reflex (Stoodley and Schmahmann, 2009; Stoodley et al., 2012). It also affects the body equilibrium during stance and gait along with lobule IX (Han et al., 2018). Thus, any change in the volume of lobule X may directly affect movement of eyes which is usually seen in ataxias (nystagmus). This finding is in agreement with the Hadjivassiliou et al. (2019) wherein they have also identified neurological deficit in terms of reduced volume of cerebellum and its IX and X lobes in the patients with celiac disease and positive for TG6 autoantibodies (Hadjivassiliou et al., 2019).

In the present study, we also observed significantly reduced volume of the thalamus in the patients with GA patients compared to healthy controls. The thalamus plays an important role in motor control and acts as a relay center between the cerebellum and the motor cortex (Sommer, 2003). The volume loss in the thalamic subnuclei has also been reported in patients with SCA type 7 (Magon et al., 2020). The patients with CeD with TG6 autoantibodies also showed a reduced volume of the thalamus which is in agreement with our findings. The significant reduction in the volume of the thalamus in GA might be attributed to loss or impairment of TG6+ neurons, potentially affecting GABA-ergic inhibitory pathways, which is supported by brain hyperexcitability reported in the patients with celiac disease (Sarrigiannis et al., 2014).

Our results also showed significantly reduced white matter of cerebrum in the patients with GA in comparison to healthy controls. This is the first report showing the atrophy of the cerebrum in GA patients. It has been reported that 40% of the patients with GA have sensory (cerebrum) ataxia rather than cerebellar ataxia and showed no atrophy of cerebellum on MRI (Bürk et al., 2001). The role of the cerebrum is to initiate and coordinate movement, thus, any change in the volume of the cerebrum can directly affect the senses and movement. Thus, it remains to be elucidated whether any change in volume of the cerebrum in GA patient is contributing to difficulty in movement. The cerebrum is also thought to regulate the sensory function of the body and is also responsible for memory, speech and emotional response (Abhang et al., 2016). Atrophy in this region may be asymptomatic and could cause memory loss or gait abnormalities (Deutsch and Deangelis, 2014). Thus, the results of the present study depicted significant changes in the volume of the white matter of the cerebrum as well as of the cerebellum in the GA patients. Due to the low sample size in the present study, it further needs to be validated whether GA is pure cerebellar or sensory in nature.

Further, our results showed a significant increase in the volume of lateral ventricles in the patients with GA in comparison to healthy controls. Lateral ventricles are a “C” shaped cavity located one on each side of the cerebral hemisphere. Continuous enlargement in the lateral ventricles has been observed due to cerebral involution through the lifespan (Dima et al., 2021), as the age increases, or due to cell death in the adjacent brain structures (Gupta, 2017). Thus, enlargement of ventricles in the patients with GA in our study might also be indicative of gluten-mediated neurodegeneration.

Further, our MRS data revealed significant neurochemical changes in the patients with GA supporting the neurodegeneration in them compared to healthy controls. Our data showed a significantly lower level of tNAA in the right cerebellum as well as in the vermis of GA patients as compared to HCs. NAA is a neuronal health marker (Wilkinson et al., 2005), which is mainly confined to neuronal cell bodies and axons. NAA has also been implicated to serve as an osmolyte and also acts as a precursor for the synthesis of the myelin sheath of oligodendrocytes by providing acetate (Machová et al., 2009). Thus, a reduced level of tNAA in the cerebellum of GA patients might be attributed to cell dysfunction as well as neuronal loss. These findings are in agreement with an earlier MRS study on GA patients, wherein they reported lower levels of NAA/Cr and NAA/Cho in them in comparison to healthy controls (Wilkinson et al., 2005). It has also been shown that a strict gluten-free diet can lead to improvement in NAA/Cr ratio in patients with GA (Hadjivassiliou et al., 2002).

Our results also indicated a significantly lower concentration of membrane precursors GPC and PC in the vermis of GA patients, which is consistent with another recent study on ataxias of SCA1, 2, and 3 patients (Krahe et al., 2020). Choline readily gets transferred through the blood-brain barrier through three different types of choline transporters (CTLs). Expression of these CTLs was seen to be increased at the time of nerve regeneration suggesting the role of choline and CTLs in proliferation and repair (Che et al., 2002). A decrease in the concentration of these metabolites may be attributed to the decreased membrane synthesis in the cerebellar region or it can be due to the mutations in choline transporters (CTLs) (Che et al., 2002).

We have also determined the weighted measures of the metabolite concentrations for relative proportions of GM and WM in the MRS voxel. The analysis improved the statistical results. Accordingly, the weighted measures of tCr were found to be lower in the vermis of GA patients as compared to HC. Creatine (methyl guanidino-acetic acid) is an amino acid, essential for the energy metabolism of cells and neurons (Adriano et al., 2010). The function of creatine and its phosphorylated form (phosphocreatine) is to move the ATP from the site of synthesis (mitochondrion) to the sites where it is utilized. It provides rapid energy to replenish the depleting energy reserves (Beard and Braissant, 2010). Creatine also has antioxidant properties which are beneficial in degenerative diseases (e. g., ataxia) through protection against energy depletion and oxidant species (Curt et al., 2015). Our results showed that tCr was significantly decreased in the vermis of GA patients which may probably be related to the atrophy and may also indicate that the neurons are susceptible to rapid energy depletion in these patients.

We have also worked out a correlation between neurochemicals in the cerebellum and vermis with the volumetric changes in the brain of GA patients. Interestingly, we found a significant correlation between the concentration of tNAA in both vermis and cerebellum and tCho (vermis) with the volume changes in the lobule X of the cerebellum. Thus, the present study has provided a comprehensive structural characterization of cerebral degeneration in patients with GA, which has been also demonstrated by cerebral neurochemical abnormalities. The lower levels of tNAA have suggested neurodegeneration while lower tCho levels may probably be indicative of the impairment of regeneration and repair activities in the GA patients.

To the best of our knowledge, this is the first MRI and MRS-based study in the same cohort of GA patients in the Indian population. In the present study, both MRI and MRS confirmed the presence of cerebellar as well as cerebrum-related volume loss and significantly altered concentrations of different neurochemicals. The neurochemicals tNAA and tCho may have the potential to serve as early indicators of neuronal damage. Further, our findings suggested that knowledge of voxel composition of GM, WM, and CSF may help in avoiding partial volume variations in MRS, particularly in neurodegenerative diseases (Mato Abad et al., 2014). Our study has a few limitations. One of the limitations of this study is the low sample size. A similar study with a large number of patients is still warranted that may provide a better insight into the pathophysiology of GA, accurate and early diagnosis and may also help in better clinical management. Further, our study has examined the neurochemical abnormalities in the deep white matter, which may reflect the downstream effects of the cortical abnormalities.

## Conclusions

The present study revealed the neurochemical profile of vermis and right cerebellum and structural changes in various brain regions of patients with GA compared with healthy controls. The concentration of neurometabolites, tNAA, tCho and tCr were significantly lower in vermis and concentration of tCho was significantly reduced in the cerebellum regions in the patients with gluten ataxia compared to healthy controls. The patients with GA also had a significant reduction in the white matter of various brain regions and they also showed a significant reduction in the volume of lateral ventricles and thalamus compared to healthy controls. Thus, the present study has elucidated cerebral degeneration in patients with GA, which has been also demonstrated by cerebral neurochemical abnormalities. The neurochemicals (tNAA, tCho) and structural changes may serve as an indicator of cerebral damage in GA. Though our preliminary study has demonstrated several significant observations related to the pathophysiology of GA in the Indian population, however, study needs to be carried out in a greater number of patients. Further, our study showed that the weighted measures of the metabolite concentrations for relative proportions of GM and WM in the MRS voxel improved the statistical results.

## Data Availability Statement

The original contributions presented in the study are included in the article/supplementary material, further inquiries can be directed to the corresponding author/s.

## Ethics Statement

The studies involving human participants were reviewed and approved by Institute Ethics Committee, All India Institute of Medical Sciences, New Delhi. The patients/participants provided their written informed consent to participate in this study.

## Author Contributions

US: conceptualization, design of the study, final editing of the manuscript, figures, and funding. GM and AS: conceptualization and patient recruitment. VR: data acquisition, analysis, figures, and writing the original draft. IS and RT: editing the draft. PD: pathology. All authors: writing—review and editing. All authors contributed to the article and approved the submitted version.

## Conflict of Interest

The authors declare that the research was conducted in the absence of any commercial or financial relationships that could be construed as a potential conflict of interest.

## Publisher's Note

All claims expressed in this article are solely those of the authors and do not necessarily represent those of their affiliated organizations, or those of the publisher, the editors and the reviewers. Any product that may be evaluated in this article, or claim that may be made by its manufacturer, is not guaranteed or endorsed by the publisher.
